# Multicenter development and validation of machine-learning risk models to predict procedural complete revascularization and in-hospital heart failure in STEMI patients treated with primary PCI

**DOI:** 10.3389/fcvm.2026.1824937

**Published:** 2026-05-13

**Authors:** Yumin Lin, Yufeng Qin, Kangkang Ou, Jichong Zhu, Bizhi Liao

**Affiliations:** 1 Department of Cardiology, Hezhou People’s Hospital, Hezhou, China; 2 Department of Trauma Orthopaedics and Hand Surgery, Nanxishan Hospital of Guangxi Zhuang Autonomous Region (The Second People’s Hospital of Guangxi Zhuang Autonomous Region), Guilin, China; 3 Department of Cardiology, The First Affiliated Hospital of Guangxi Medical University, Nanning, China

**Keywords:** complete revascularization, in-hospital heart failure, machine learning, primary percutaneous coronary intervention (PPCI), ST-segment elevation myocardial infarction (STEMI)

## Abstract

**Background:**

In-hospital heart failure (HF) remains common after primary percutaneous coronary intervention (PPCI) for ST-segment elevation myocardial infarction (STEMI) and is associated with adverse in-hospital outcomes. In addition, whether procedural complete revascularization (CR) can be achieved during the index PCI is clinically relevant but often constrained in real-world practice. We aimed to develop and externally validate machine-learning (ML) models for these two complementary prediction tasks.

**Methods:**

We conducted a multicenter cohort study of STEMI patients treated with PPCI from three hospitals. Patients from Hezhou People's Hospital (January 2020 to June 2024) comprised the training cohort (*n* = 734). Patients from two other centers (July 2024 to December 2025) were combined as an independent testing cohort (*n* = 352). Multiple ML algorithms were benchmarked to predict (1) in-hospital HF and (2) the real-world feasibility of achieving procedural CR during the index PCI. Model performance was assessed using the area under the receiver operating characteristic curve (AUC), the area under the precision-recall curve (AUPRC), classification metrics, calibration curves, decision curve analysis (DCA), and clinical impact curves. Shapley Additive Explanations (SHAP) were used to enhance interpretability.

**Results:**

For in-hospital HF prediction, CatBoost showed the best overall performance in the independent testing cohort (AUC: 0.973; 95% CI: 0.957–0.989; accuracy: 88.6%), with good calibration and favorable net benefit on DCA. For procedural CR prediction, CatBoost was also selected as the primary model based on its overall performance profile in the independent testing cohort (AUC: 0.970; 95% CI: 0.954–0.987; accuracy: 92.0%), with acceptable calibration and positive net benefit across a broad range of threshold probabilities. Key predictors included LAD involvement, age, symptom-to-guidewire crossing time, and markers related to inflammation, coagulation, renal function, and lipid metabolism.

**Conclusions:**

In a three-center cohort, we developed and externally validated two ML models for predicting subsequent in-hospital HF after index PPCI and the feasibility of achieving procedural CR during the index PCI. Both models demonstrated good discrimination, calibration, clinical utility, and interpretability, supporting peri-procedural risk stratification and catheterization-laboratory decision support in STEMI patients treated with PPCI.

## 1 Introduction

Although primary percutaneous coronary intervention (PPCI) has become the standard reperfusion strategy for ST-segment elevation myocardial infarction (STEMI), in-hospital heart failure (HF) remains relatively common and is closely associated with adverse in-hospital outcomes (1). Prior studies have shown that HF during the acute phase of STEMI—often reflected by higher Killip class—is not uncommon; moreover, HF present on admission or developing during hospitalization is consistently linked to a higher risk of in-hospital events and mortality, and frequently necessitates higher-acuity monitoring and supportive therapies, thereby increasing healthcare resource utilization (2).

In STEMI patients with multivessel coronary artery disease, complete revascularization (CR) has been demonstrated in randomized trials to reduce ischemia-driven adverse outcomes (3). For example, the COMPLETE trial showed that, compared with culprit-lesion–only PCI, a CR strategy further reduced hard endpoints such as cardiovascular death or myocardial infarction, supporting an overall clinical benefit of CR (4). Accordingly, the 2023 European Society of Cardiology (ESC) guidelines for acute coronary syndromes recommend that, in hemodynamically stable STEMI patients with multivessel disease, non-culprit lesion revascularization may be performed either during the index PCI procedure or within a defined time window, whereas in patients with cardiogenic shock an infarct-related-artery–only approach is generally favored, underscoring the context-dependent nature of CR decision-making (5). However, in real-world practice, whether procedural (index-procedure) CR can be achieved is not determined by anatomy alone. It is jointly influenced by lesion complexity, ischemic burden, procedural risk, hemodynamic status, and operator judgment under time and resource constraints in the catheterization laboratory (6). Therefore, rather than modeling a purely physiological outcome, the present study aimed to develop a prediction model for the real-world likelihood/feasibility of achieving procedural CR during the index PPCI. Such a model may help inform early consideration of immediate vs. staged strategies, facilitate cath-lab workflow and resource planning, and provide a pragmatic basis for identifying patients in whom same-session CR is more or less likely to be achievable (7).

Most prior studies assessing risk in STEMI patients undergoing PPCI—either for (in-hospital) HF or for procedural CR—have been derived from single-center or highly selected cohorts, limiting generalizability and transportability. Even when regression- or machine-learning–based approaches were applied, external validation has often been insufficient and calibration performance incompletely reported, constraining the interpretability of predicted probabilities (8, 9). In addition, evaluation of clinical utility (e.g., decision-curve analysis and net benefit) remains limited, which hampers translation of these models into real-world clinical workflows (10). Recent cardiovascular prediction studies have increasingly incorporated multimodal data, interpretable modeling frameworks, and multicenter validation strategies, underscoring the growing need for clinically usable and transportable risk-prediction tools in real-world settings (11, 12).

In this multicenter study of STEMI patients treated with PPCI, we addressed two complementary prediction tasks. First, we developed and validated a risk model to predict (in-hospital) HF; second, we developed and validated a model to estimate the real-world feasibility/likelihood of achieving procedural CR during the index PCI. We systematically compared multiple ML algorithms in the training cohort and an independent testing cohort in terms of discrimination, calibration, and clinical utility, and applied explainability methods to identify and interpret the key predictors for each endpoint, thereby informing peri-procedural risk stratification and cath-lab decision support.

## 2 Methods

### 2.1 Study design & data source

We conducted a multicenter cohort study using routinely collected clinical data from three hospitals. Patients treated at Hezhou People's Hospital between January 2020 and June 2024 were included as the training cohort for model development. For independent external validation, patients from the other two centers were enrolled between July 2024 and December 2025, and the two datasets were combined to form the validation cohort. The study protocol was approved by the Ethics Committee of Hezhou People's Hospital (approval No. KY2024030605; Supplementary Material 1).

### 2.2 Participants

We consecutively enrolled STEMI patients admitted to three centers. The inclusion criteria were as follows: (1) meeting the diagnostic criteria of the 2019 guideline for the diagnosis and treatment of acute STEMI (13); and (2) receiving PPCI within 12 h of symptom onset, or undergoing PPCI beyond 12 h when there was clinical and/or electrocardiographic evidence of ongoing/progressive myocardial ischemia. The exclusion criteria were: (1) a history of chronic HF, congenital heart disease, cardiomyopathy, or severe valvular heart disease; (2) incomplete key clinical data; and (3) in-hospital death.

Baseline characteristics and candidate predictors were extracted from routinely collected clinical records, including demographic characteristics, comorbidities, admission laboratory measurements, and selected angiographic/procedural variables. For model development, only variables available up to the predefined prediction time-zero for each endpoint were considered eligible predictors.

### 2.3 Outcomes

Two relatively independent prediction endpoints were defined: (1) occurrence of (in-hospital) HF during hospitalization; and (2) achievement of CR during the index PCI. Both endpoints were adjudicated based on inpatient medical records, laboratory and imaging data, and catheterization laboratory procedural documentation.

#### 2.3.1 Definition and adjudication window for (in-hospital) HF

(In-hospital) HF was defined as the occurrence of clinically recognized acute HF/cardiac dysfunction during the index hospitalization. The endpoint was not based on administrative discharge codes alone, but was adjudicated from the inpatient clinical record using a combination of: (1) clinician-documented new-onset or worsening signs/symptoms consistent with HF or hemodynamic deterioration during hospitalization; (2) supportive imaging or echocardiographic evidence of cardiac dysfunction, when available; and/or (3) initiation or escalation of HF-directed therapies during the hospital stay, such as intravenous diuretics, inotropes, or other circulatory/respiratory supportive measures. The adjudication window spanned from admission to discharge (14, 15). In the present study, this endpoint was treated as a broader in-hospital HF occurrence outcome and was not further subdivided into HF present at admission vs. incident HF developing later during hospitalization.

#### 2.3.2 Definition and adjudication window for procedural CR (index PCI/same-session PPCI)

Procedural CR was defined as follows: after completion of the index PCI (same-session PPCI), in addition to treatment of the culprit vessel, any non-culprit lesions with significant stenosis deemed to require revascularization were also treated during the same procedure (e.g., balloon angioplasty and/or stent implantation), thereby achieving complete revascularization within the index procedure (16). Conversely, procedural CR was considered not achieved if, at the end of the index PCI, any significant non-culprit lesions remained untreated (regardless of whether staged PCI was planned during the index hospitalization), or if only culprit-lesion intervention was performed (17).

To reduce temporal ambiguity and minimize the risk of information leakage, prediction time-zero was defined separately for the two endpoints. For the (in-hospital) HF model, prediction time-zero was set at completion of the index PPCI, and the model was designed to predict HF events occurring subsequently during the remainder of hospitalization. Accordingly, eligible predictors for this task were limited to variables available at admission or during the index procedure up to completion of PPCI.

For the procedural CR model, prediction time-zero was defined before final completion of the revascularization strategy during the index PCI. Therefore, only variables available at admission and during procedural assessment up to that time point were considered eligible predictors. Variables directly reflecting subsequent outcome occurrence or clearly downstream management decisions were not intended to serve as proxies for the study outcomes.

### 2.4 Modeling strategy

For the two relatively independent endpoints—(in-hospital) HF during hospitalization and achievement of CR during the index PCI—we applied a uniform machine-learning workflow while developing and validating two separate models. The dataset was partitioned by center and time period. All candidate predictors retained after dataset curation were entered into the modeling workflow, and no additional outcome-driven univariable screening was performed at the model-building stage. We compared multiple algorithms, including Logistic regression/Lasso, support vector machine (SVM), random forest (RF), gradient boosting decision tree (GBDT), CatBoost, LightGBM, neural networks (NeuralNet), partial least squares (PLS), discriminant analysis, and Bayesian methods. Hyperparameters were tuned within the training cohort using cross-validation in combination with grid search and/or other optimization strategies, as appropriate. Predicted probabilities were converted into binary class labels using a threshold of 0.5. For models implemented within the general training framework, training-cohort performance was summarized mainly on the basis of cross-validation predictions, and these comparative results are additionally reported in the Supplementary Tables to facilitate a more robust assessment of model stability. For CatBoost and LightGBM, training-set predictions were additionally obtained directly from the fitted models; therefore, their apparent training-cohort performance was interpreted cautiously. The analytical dataset was largely complete, with no substantial missingness across the main candidate predictors. Only a small number of variables had occasional missing values, which were handled using a predefined simple imputation strategy according to variable type, with median imputation for continuous variables and mode imputation for categorical variables (18). All analyses were performed in R using the packages caret, pROC, ggplot2, shapviz, kernelshap, dplyr, rmda, catboost, and lightgbm. The main modeling workflow and software environment have been added to improve reproducibility.

In the procedural CR task, Bilateral-or-Multivessel-Disease showed disproportionately high predictive dominance. To reduce over-reliance on a single feature and to assess model robustness, we performed a sensitivity analysis by excluding this variable and repeating model training, hyperparameter tuning, and evaluation using the same workflow.

The independent testing cohort was used as the principal benchmark for model comparison and interpretation. Given the possibility of optimistic performance estimates in the training cohort, the primary analyses emphasized discrimination, calibration, and clinical utility in the independent testing cohort rather than the apparent performance in the training cohort. For the HF prediction task, the final interpretation of model behavior relied primarily on the dominant features identified by SHAP, which were mainly demographic, laboratory, ischemic-time, and angiographic/procedural variables rather than medication-related variables.

### 2.5 Performance evaluation

Model discrimination was primarily assessed using the area under the receiver operating characteristic curve (AUROC), with the area under the precision–recall curve (AUPRC) additionally reported. Classification performance metrics included accuracy, F1-score, positive predictive value (PPV), negative predictive value (NPV), sensitivity, specificity, and the Youden index. Calibration was evaluated using calibration curves. Clinical utility was assessed using decision curve analysis (DCA) and clinical impact curves to quantify net benefit across a range of threshold probabilities and to illustrate potential clinical value.

### 2.6 Explainability

For the best-performing models, we used Shapley Additive Explanations (SHAP) to enhance interpretability. Global feature importance and SHAP beeswarm plots were used to identify key predictors and their directional contributions, while SHAP dependence plots were generated to depict the relationships between pivotal features and model outputs. In addition, individual-level explanations were provided to support clinical interpretability and facilitate potential implementation (19).

## 3 Results

### 3.1 Patient selection and cohort characteristics

**Figure 1:**
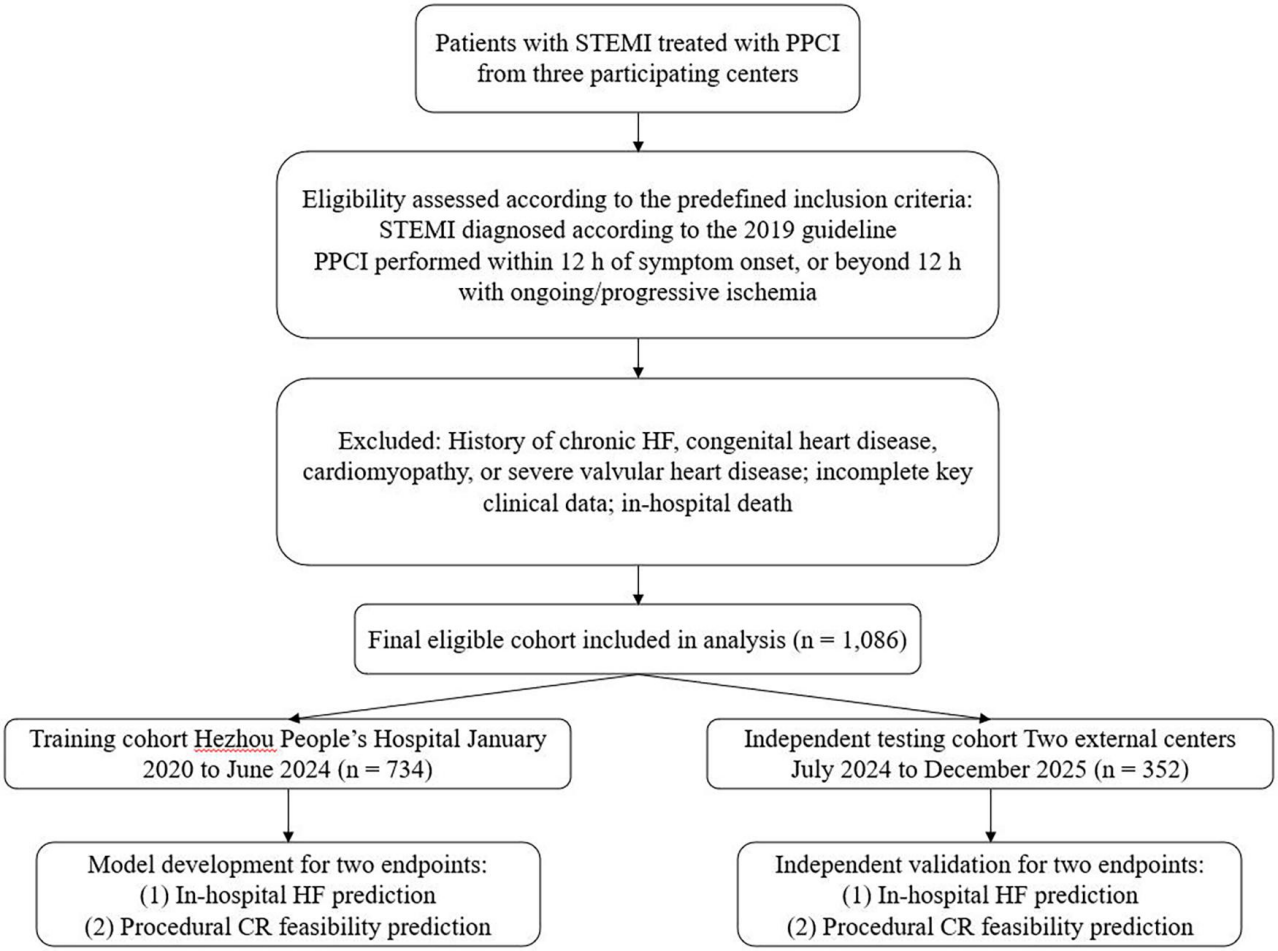
Flowchart showing the screening and selection of STEMI patients treated with PPCI from three participating centers, including application of the predefined inclusion and exclusion criteria and assignment to the training cohort (*n* = 734) and independent testing cohort (*n* = 352). Both endpoints—(in-hospital) HF prediction and procedural CR feasibility prediction—were modeled using the same training/testing framework.

The patient selection process is shown in Figure 1. After application of the predefined inclusion and exclusion criteria, a total of 1,086 eligible STEMI patients treated with PPCI were included in the final analysis, comprising 734 patients in the training cohort and 352 patients in the independent testing cohort. Baseline characteristics were well balanced between the two cohorts (Table 1). The two cohorts showed similar distributions of demographic features (e.g., age and gender), comorbidities, laboratory indices, and other clinical/procedural variables, with no statistically significant differences observed across baseline variables (all *P* > 0.05). These findings support the comparability of the cohorts and the suitability of the testing cohort for independent validation. The same training/testing cohort partition was applied to both prediction tasks, namely (in-hospital) HF prediction and procedural CR feasibility prediction.

**Table 1:** Baseline characteristics and outcome-related differences of all variables in the training and testing cohorts.

Variables	Training Cohort (*n* = 734)	Validation Cohort (*n* = 352)	*P*-value
Demographics			
Gender (male, %)	462 (62.9)	218 (61.9)	0.748
Age (years)	63.8 ± 10.4	64.1 ± 10.1	0.655
Smoking (%)	182 (24.8)	86 (24.4)	0.902
Comorbidities			
Diabetes (%)	218 (29.7)	104 (29.5)	0.954
Hypertension (%)	412 (56.1)	196 (55.7)	0.887
COPD (%)	38 (5.2)	19 (5.4)	0.875
Atrial Fibrillation (%)	42 (5.7)	20 (5.7)	0.985
Malignant Neoplasms (%)	15 (2.0)	7 (2.0)	0.965
Previous MI (%)	62 (8.4)	28 (7.9)	0.784
Previous Coronary Revasc (%)	45 (6.1)	21 (6.0)	0.912
CKD (%)	52 (7.1)	25 (7.1)	0.988
Laboratory			
WBC (10^9^/L)	7.6 ± 2.1	7.5 ± 2.2	0.482
NEUT (10^9^/L)	5.2 ± 1.8	5.1 ± 1.9	0.415
Hb (g/L)	132 ± 15	133 ± 14	0.312
PLT (10^9^/L)	215 ± 62	218 ± 58	0.455
hsCRP (mg/L)	3.8 (1.2, 9.5)	3.9 (1.3, 9.8)	0.612
Cholesterol (mmol/L)	4.2 ± 0.9	4.1 ± 1.0	0.125
Triglycerides (mmol/L)	1.6 (1.1, 2.2)	1.7 (1.2, 2.3)	0.245
HDL-C (mmol/L)	1.12 ± 0.25	1.10 ± 0.28	0.255
LDL-C (mmol/L)	2.65 ± 0.85	2.62 ± 0.82	0.589
ALB (g/L)	40.2 ± 4.5	39.8 ± 4.8	0.189
UA (umol/L)	365 ± 82	362 ± 85	0.589
BUN (mmol/L)	5.8 (4.2, 8.5)	5.9 (4.3, 8.6)	0.674
D-Dimer (mg/L)	0.45 (0.2, 1.2)	0.48 (0.2, 1.3)	0.512
Angiographic			
Time to Guidewire (h)	6.2 ± 2.5	6.4 ± 2.3	0.218
LAD Occlusion (%)	352 (48.0)	168 (47.7)	0.942
LCX Occlusion (%)	185 (25.2)	90 (25.6)	0.892
RCA Occlusion (%)	197 (26.8)	94 (26.7)	0.965
Bilateral/Multivessel (%)	245 (33.4)	116 (33.0)	0.885
Overall complete revascularization during index hospitalization (%)	410 (55.9)	198 (56.2)	0.912
Procedural			
Postoperative Slow Flow (%)	56 (7.6)	26 (7.4)	0.895
Postoperative No Reflow (%)	22 (3.0)	11 (3.1)	0.925
Medication			
ACEI ARB ARNI (%)	315 (42.9)	152 (43.2)	0.932
Beta blockers (%)	450 (61.3)	215 (61.1)	0.944

Bold values indicate statistical significance (*P* < 0.05).

Baseline demographic, clinical, laboratory, and procedural variables were compared between the training cohort (Hezhou People's Hospital, January 2020–June 2024; *n* = 734) and the independent testing cohort (two external centers, July 2024–December 2025; *n* = 352). Continuous variables are presented as mean (standard deviation) or median (interquartile range), as appropriate; categorical variables are presented as *n* (%). *P* values were calculated using appropriate statistical tests according to data type and distribution.

COPD, chronic obstructive pulmonary disease; MI, myocardial infarction; CKD, chronic kidney disease; WBC, white blood cell count; NEUT, neutrophil count; Hb, hemoglobin; PLT, platelet count; hsCRP, high-sensitivity C-reactive protein; HDL-C, high-density lipoprotein cholesterol; LDL-C, low-density lipoprotein cholesterol; ALB, albumin; UA, uric acid; BUN, blood urea nitrogen; LAD, left anterior descending artery; LCX, left circumflex artery; RCA, right coronary artery; ACEI, angiotensin-converting enzyme inhibitor; ARB, angiotensin receptor blocker; ARNI, angiotensin receptor–neprilysin inhibitor.

### 3.2 Outcome 1: in-hospital HF prediction model

#### 3.2.1 Comparison of HF and Non-HF baselines

Across both the training cohort (Table 2) and the testing cohort (Table 3), baseline characteristics showed a consistent pattern of differences between the HF and non-HF groups. Compared with the non-HF group, patients who developed HF were older, more frequently had LAD-related lesions, and had a longer symptom-to-guidewire crossing time (Time to Guidewire) (both cohorts, *P* < 0.001), indicating that prolonged ischemic time and delayed reperfusion may contribute to a higher risk of (in-hospital) HF. In terms of coronary anatomy, the HF group had a higher prevalence of Bilateral or Multivessel Disease (both cohorts, *P* < 0.001), further indicating a greater overall atherosclerotic burden.

**Table 2:** Comparison of baseline characteristics between HF and Non-HF patients in the training cohort.

Variables	HF (*n* = 307)	Non-HF (*n* = 427)	*P*-value
Demographics			
Gender (male, %)	198 (64.5%)	272 (63.7%)	0.824
Age (years)	69.12 ± 9.45	60.23 ± 10.12	**<0.001**
Smoking (%)	78 (25.4%)	105 (24.6%)	0.798
Comorbidities			
Diabetes (%)	115 (37.5%)	82 (19.2%)	**<0.001**
Hypertension (%)	205 (66.8%)	195 (45.7%)	**<0.001**
COPD (%)	22 (7.2%)	14 (3.3%)	**0.015**
Atrial Fibrillation (%)	28 (9.1%)	12 (2.8%)	**<0.001**
Malignant Neoplasms (%)	6 (2.0%)	8 (1.9%)	0.945
Previous MI (%)	35 (11.4%)	22 (5.2%)	**0.002**
Previous Coronary Revasc (%)	22 (7.2%)	18 (4.2%)	0.078
CKD (%)	38 (12.4%)	15 (3.5%)	**<0.001**
Laboratory			
WBC (10^9^/L)	9.24 ± 2.85	6.45 ± 1.92	**<0.001**
NEUT (10^9^/L)	6.52 ± 2.15	4.18 ± 1.45	**<0.001**
Hb (g/L)	122.5 ± 18.4	138.2 ± 14.2	**<0.001**
PLT (10^9^/L)	215 ± 68	212 ± 62	0.542
hsCRP (mg/L)	9.85 (4.21, 22.45)	1.52 (0.65, 3.24)	**<0.001**
Cholesterol (mmol/L)	4.28 ± 1.12	4.15 ± 0.88	0.082
Triglycerides (mmol/L)	1.82 (1.25, 2.12)	0.012
HDL-C (mmol/L)	1.02 ± 0.21	1.18 ± 0.28	**<0.001**
LDL-C (mmol/L)	2.88 ± 0.94	2.42 ± 0.75	**<0.001**
ALB (g/L)	36.52 ± 5.12	42.48 ± 3.65	**<0.001**
UA (umol/L)	412 ± 95	328 ± 72	**<0.001**
BUN (mmol/L)	8.82 (5.45, 13.12)	4.55 (3.52, 6.28)	**<0.001**
D-Dimer (mg/L)	1.45 (0.68, 3.25)	0.22 (0.11, 0.42)	**<0.001**
Angiographic			
Time to Guidewire (h)	8.95 ± 3.42	4.12 ± 1.85	**<0.001**
LAD Occlusion (%)	188 (61.2%)	155 (36.3%)	**<0.001**
LCX Occlusion (%)	82 (26.7%)	108 (25.3%)	0.672
RCA Occlusion (%)	88 (28.7%)	112 (26.2%)	0.456
Bilateral/Multivessel (%)	142 (46.3%)	95 (22.2%)	**<0.001**
Overall complete revascularization during index hospitalization (%)	145 (47.2%)	265 (62.1%)	**<0.001**
Procedural			
Postoperative Slow Flow (%)	45 (14.7%)	12 (2.8%)	**<0.001**
Postoperative No Reflow (%)	15 (4.9%)	5 (1.2%)	**0.002**
Medication			
ACEI ARB ARNI (%)	135 (44.0%)	182 (42.6%)	0.715
Beta blockers (%)	192 (62.5%)	255 (59.7%)	0.442

Bold values indicate statistical significance (*P* < 0.05).

Baseline variables were compared between patients who developed (in-hospital) HF and those without (in-hospital) HF in the training cohort (*n* = 734). Data presentation and statistical testing are as described in Table 1.

HF, heart failure; LAD, left anterior descending artery; Time-to-Guidewire, symptom-to-guidewire crossing time; other abbreviations as in Table 1.

**Table 3:** Baseline characteristics stratified by in-hospital HF status in the testing cohort.

Variables	HF (*n* = 171)	Non-HF (*n* = 181)	*P*-value
Demographics			
Gender (male, %)	108 (63.2%)	110 (60.8%)	0.648
Age (years)	69.45 ± 8.92	60.84 ± 9.85	**<0.001**
Smoking (%)	43 (25.1%)	45 (24.9%)	0.952
Comorbidities			
Diabetes (%)	64 (37.4%)	41 (22.7%)	**<0.001**
Hypertension (%)	115 (67.3%)	82 (45.3%)	**<0.001**
COPD (%)	12 (7.0%)	7 (3.9%)	0.185
Atrial Fibrillation (%)	15 (8.8%)	5 (2.8%)	**0.012**
Malignant Neoplasms (%)	3 (1.8%)	4 (2.2%)	0.752
Previous MI (%)	19 (11.1%)	9 (5.0%)	**0.035**
Previous Coronary Revasc (%)	11 (6.4%)	10 (5.5%)	0.724
CKD (%)	21 (12.3%)	4 (2.2%)	**<0.001**
Laboratory			
WBC (10^9^/L)	9.32 ± 2.62	6.55 ± 1.85	**<0.001**
NEUT (10^9^/L)	6.58 ± 2.12	4.25 ± 1.38	**<0.001**
Hb (g/L)	121.8 ± 17.5	138.5 ± 13.8	**<0.001**
PLT (10^9^/L)	216 ± 62	219 ± 58	0.645
hsCRP (mg/L)	9.92 (4.18, 22.15)	1.55 (0.68, 3.22)	**<0.001**
Cholesterol (mmol/L)	4.35 ± 1.08	4.12 ± 0.92	0.065
Triglycerides (mmol/L)	1.85 (1.28, 2.52)	1.52 (1.02, 2.15)	**0.008**
HDL-C (mmol/L)	1.03 ± 0.22	1.16 ± 0.25	**<0.001**
LDL-C (mmol/L)	2.92 ± 0.91	2.45 ± 0.72	**<0.001**
ALB (g/L)	36.45 ± 5.02	42.68 ± 3.55	**<0.001**
UA (umol/L)	415 ± 92	331 ± 68	**<0.001**
BUN (mmol/L)	8.92 (5.52, 13.24)	4.62 (3.55, 6.32)	**<0.001**
D-Dimer (mg/L)	1.48 (0.72, 3.18)	0.24 (0.12, 0.44)	**<0.001**
Angiographic			
Time to Guidewire (h)	8.98 ± 3.52	4.18 ± 1.88	**<0.001**
LAD Occlusion (%)	105 (61.4%)	63 (34.8%)	**<0.001**
LCX Occlusion (%)	45 (26.3%)	46 (25.4%)	0.842
RCA Occlusion (%)	50 (29.2%)	48 (26.5%)	0.565
Bilateral/Multivessel (%)	78 (45.6%)	42 (23.2%)	**<0.001**
Overall complete revascularization during index hospitalization (%)	82 (48.0%)	112 (61.9%)	**0.008**
Procedural			
Postoperative Slow Flow (%)	26 (15.2%)	5 (2.8%)	**<0.001**
Postoperative No Reflow (%)	9 (5.3%)	2 (1.1%)	**0.022**
Medication			
ACEI ARB ARNI (%)	75 (43.9%)	77 (42.5%)	0.812
Beta blockers (%)	108 (63.2%)	111 (61.3%)	0.724

Bold values indicate statistical significance (*P* < 0.05).

Baseline variables were compared between patients who developed (in-hospital) HF and those without (in-hospital) HF in the independent testing cohort (*n* = 352). Data presentation and statistical testing are as described in Table 1.

HF, heart failure; LAD, left anterior descending artery; Time-to-Guidewire, symptom-to-guidewire crossing time; other abbreviations as in Table 1.

Regarding laboratory findings, the HF group demonstrated significantly higher levels of BUN, D-Dimer, WBC, NEUT, UA, and hsCRP (both cohorts, *P* < 0.001), whereas Hb, HDL-C, and ALB were significantly lower (both cohorts, *P* < 0.001). These differences collectively reflect a more adverse systemic profile characterized by impaired renal function and increased metabolic burden, activation of the coagulation–fibrinolysis system, heightened inflammatory response, and poorer nutritional/synthetic status. In addition, Cholesterol (TC) also differed between the two groups (as shown in the tables), suggesting that lipid status may be associated with HF risk stratification.

#### 3.2.2 Comparison of model performance (training and testing cohorts)

**Figure 2:**
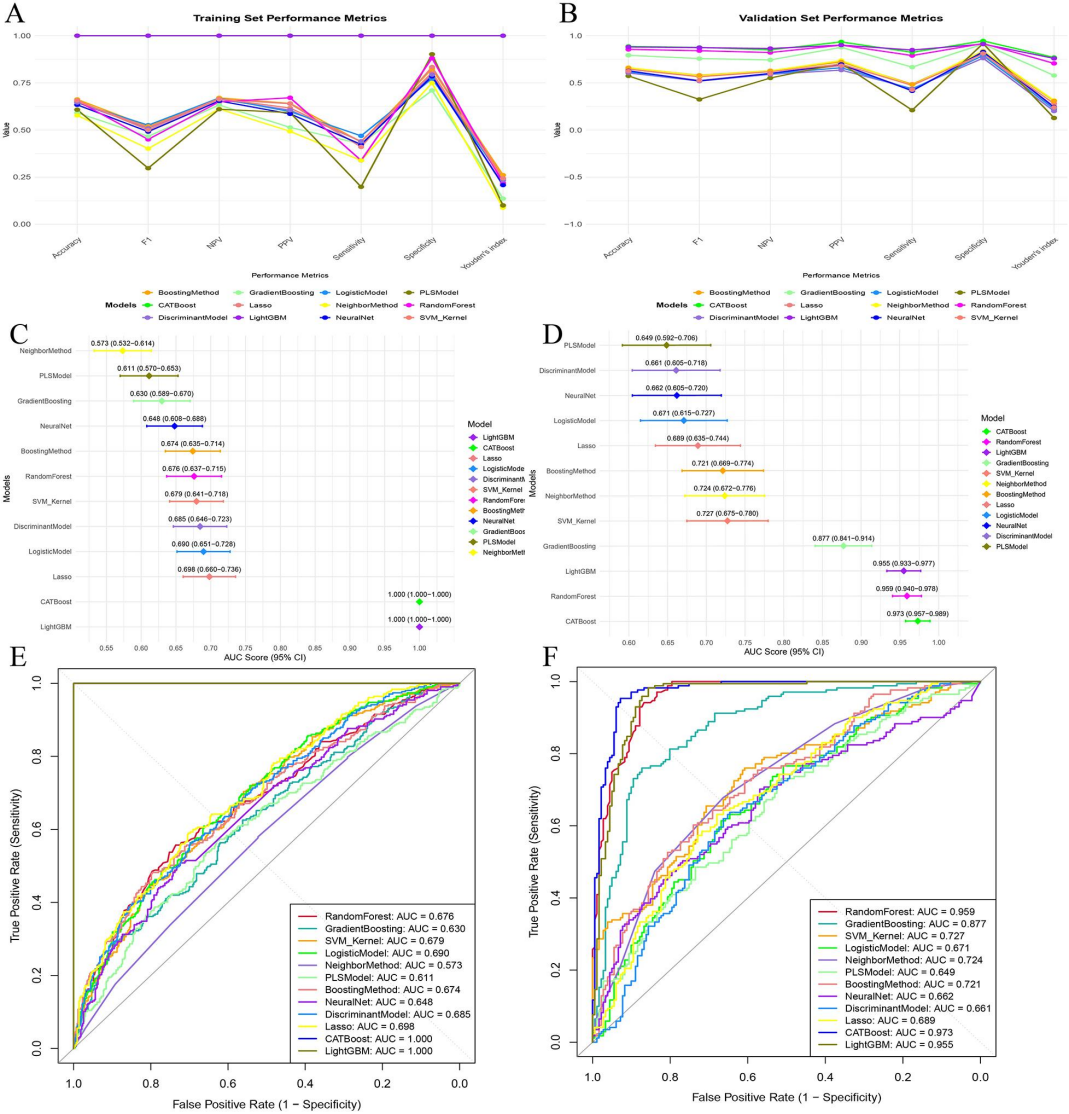
Model performance comparison for (in-hospital) HF prediction in the training and testing cohorts. **(A)** Training-cohort performance metrics across machine-learning models, including accuracy, F1-score, NPV, PPV, sensitivity, specificity, and Youden index. **(B)** Testing-cohort performance metrics across models (the “validation set” in the figure corresponds to the independent testing cohort). **(C)** Training-cohort AUCs with 95% confidence intervals across models. **(D)** Testing-cohort AUCs with 95% confidence intervals across models. **(E)** Training-cohort ROC curves across models. **(F)** Testing-cohort ROC curves across models. The corresponding metric values are labeled in the plot for clarity. HF, heart failure; AUC, area under the receiver operating characteristic curve; ROC, receiver operating characteristic; PPV, positive predictive value; NPV, negative predictive value.

For the (in-hospital) HF prediction task, we systematically compared the performance of multiple ML models in the training cohort and the independent testing cohort (Figures 2A–F). Overall, the multi-metric comparisons showed substantial heterogeneity across algorithms. In both cohorts, most candidate models tended to exhibit relatively higher specificity and NPV than sensitivity, indicating that several models were better at ruling out HF than identifying all event cases (Figures 2A,B).

In the training cohort, CatBoost and LightGBM showed the most favorable overall performance across the combined classification metrics, including accuracy, F1-score, PPV, NPV, sensitivity, specificity, and the Youden index (Figure 2A). Their apparent discrimination was also highest in the AUC forest plot and ROC analysis (Figures 2C,E). By contrast, the remaining models showed clearly lower and more variable performance, indicating that predictive ability was strongly model-dependent rather than uniformly high across algorithms.

In the independent testing cohort, the between-model ranking remained generally consistent, although performance decreased compared with the training cohort (Figures 2B–F). CatBoost achieved the highest discrimination (AUC = 0.973; 95% CI: 0.957–0.989), followed by RandomForest (AUC = 0.959; 95% CI: 0.940–0.978) and LightGBM (AUC = 0.955; 95% CI: 0.933–0.977). GradientBoosting also retained acceptable discrimination (AUC = 0.877; 95% CI: 0.841–0.914), whereas several other candidate models showed only modest performance. The ROC curves further confirmed the advantage of the leading tree-based models over the remaining algorithms (Figure 2F).

The complete comparative performance metrics of all candidate models in the training and independent testing cohorts are additionally provided in Supplementary Table 1. Taken together, considering the complete performance profile in the independent testing cohort—including AUC, accuracy, specificity, PPV, F1-score, and the Youden index—CatBoost demonstrated the most favorable overall performance and was therefore selected for subsequent analyses. The exact comparative performance values of all candidate models in the training and independent testing cohorts are provided in Supplementary Table 1.

#### 3.2.3 Clinical utility and calibration of the best-performing model

**Figure 3:**
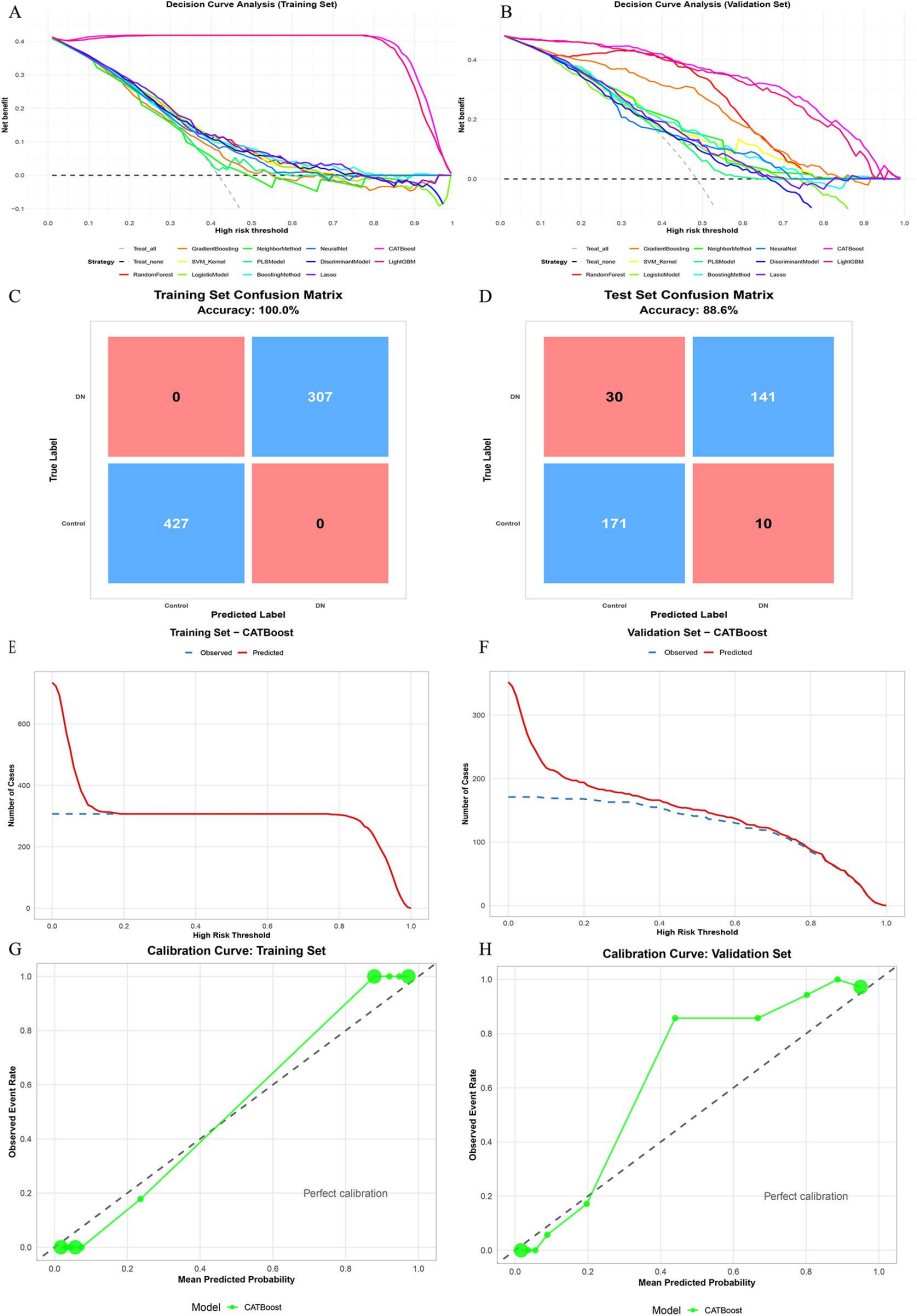
Clinical utility, classification performance, and calibration of the catBoost model for (in-hospital) HF prediction. **(A)** DCA of candidate models in the training cohort. **(B)** DCA of candidate models in the testing cohort. **(C)** Confusion matrix of the CatBoost model in the training cohort. **(D)** Confusion matrix of the CatBoost model in the testing cohort. **(E)** Clinical impact curve of the CatBoost model in the training cohort (predicted high-risk vs observed events across threshold probabilities). **(F)** Clinical impact curve of the CatBoost model in the testing cohort. **(G)** Calibration curve of the CatBoost model in the training cohort. **(H)** Calibration curve of the CatBoost model in the testing cohort. DCA, decision curve analysis; HF, heart failure.

After selecting CatBoost as the primary model, we further evaluated its clinical utility and calibration in the training cohort and the independent testing cohort (Figures 3A–H). In DCA, CatBoost achieved a favorable net-benefit profile and outperformed the “treat-all” and “treat-none” strategies in both cohorts, indicating potential clinical usefulness (Figures 3A,B). The confusion matrices illustrate the classification patterns of the fitted model in the training and testing cohorts (Figures 3C,D). Although the training-cohort confusion matrix showed apparent perfect classification, this result was interpreted cautiously and not taken as evidence of true generalizability. By contrast, the independent testing cohort showed an accuracy of 88.6%, with 30 HF cases predicted as non-HF and 10 non-HF cases predicted as HF. The clinical impact curves further demonstrated that, across varying high-risk thresholds, the number of individuals classified as high risk and the corresponding number of observed events followed concordant trends, supporting the practical interpretability of risk stratification (Figures 3E,F). Calibration curves indicated good agreement between predicted probabilities and observed event rates in the training cohort. In the independent testing cohort, the calibration plot also showed an overall acceptable correspondence between predicted and observed event probabilities, although some visual departure from the ideal reference line was observed in the intermediate-probability range (Figures 3G,H). This observation was interpreted descriptively on the basis of the calibration plot and was not intended to imply formal statistical significance of local deviation. Overall, the model maintained good discrimination and favorable net benefit in the testing cohort. Complete comparative performance metrics, including training-framework results and independent testing-cohort results across candidate models, are additionally provided in Supplementary Table 1.

#### 3.2.4 Explainability analysis

**Figure 4:**
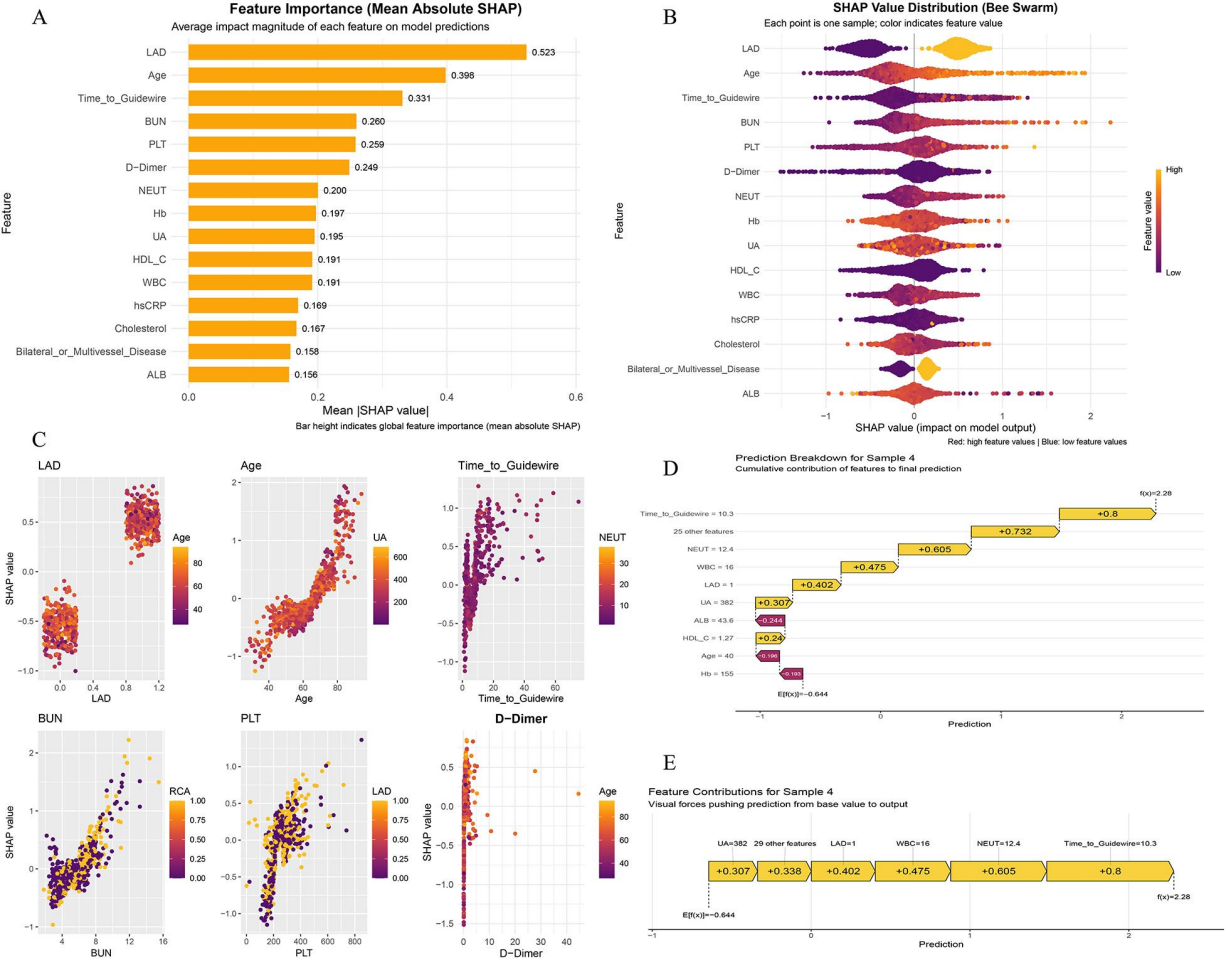
SHAP-based interpretability of the catBoost model for (in-hospital) HF prediction. **(A)** Global feature importance ranked by mean absolute SHAP values. **(B)** SHAP beeswarm plot showing the distribution and directionality of feature effects across individuals (color indicates feature value). **(C)** SHAP dependence plots for selected key predictors illustrating potentially non-linear associations with the model output. **(D)** Prediction breakdown (waterfall plot) for one representative patient selected from the training cohort, showing how features cumulatively shift the prediction from the baseline (expected) value to the final output. **(E)** Feature contribution (force-like) plot for the same representative patient from the training cohort.

After model selection, we used SHAP to interpret the best-performing CatBoost model (Figures 4A–E). Global SHAP feature importance indicated that LAD, Age, Time-to-Guidewire, BUN, PLT, D-Dimer, NEUT, Hb, UA, HDL-C, and WBC were among the most influential predictors (Figure 4A), and the SHAP beeswarm plot further illustrated the directional effects of these features across their value ranges (Figure 4B). SHAP dependence plots suggested that older Age, a longer symptom-to-guidewire crossing time (Time-to-Guidewire), higher BUN/D-Dimer/WBC/NEUT/UA, and lower Hb/HDL-C (as well as selected nutritional markers such as ALB) were generally associated with higher SHAP values, thereby pushing the model toward a higher predicted risk of (in-hospital) HF (Figure 4C). Notably, the dominant contributors in the final SHAP analysis were mainly demographic, laboratory, ischemic-time, and angiographic/procedural variables, whereas medication-related variables were not among the principal drivers of model output. In addition, ACEI/ARB/ARNI and beta blockers did not show statistically significant differences between the HF and non-HF groups in either cohort, suggesting that they were unlikely to be major drivers of the observed model discrimination. At the individual level, we present the explanation for one representative case selected from the training cohort in Figures 4D,E to illustrate how specific features cumulatively increased or decreased the final prediction; explanations for the remaining cases are provided in Supplementary Figure 1.

### 3.3 Outcome 2: prediction of procedural complete revascularization feasibility

#### 3.3.1 Complete vs. incomplete revascularization (CR vs. incomplete)

Baseline characteristics stratified by revascularization status are summarized in the training cohort (Table 4: CR *n* = 346 vs. incomplete *n* = 388) and the testing cohort (Table 5: CR *n* = 165 vs. incomplete *n* = 187). Overall, a consistent pattern of differences was observed across cohorts. Compared with the CR group, the incomplete group more frequently had LAD-related lesions and exhibited a markedly longer symptom-to-guidewire crossing time (Time-to-Guidewire) (both cohorts, *P* < 0.001), suggesting a greater ischemic burden and more pronounced delay in reperfusion among patients who did not achieve procedural CR.

**Table 4:** Baseline characteristics stratified by procedural CR status in the training cohort.

Variables	Complete Revascularization (*n* = 346)	Incomplete Revascularization (*n* = 388)	*P*-value
Demographics			
Gender (male, %)	225 (65.0%)	248 (63.9%)	0.758
Age (years)	62.45 ± 10.12	64.12 ± 9.85	0.056
Smoking (%)	85 (24.6%)	102 (26.3%)	0.598
Comorbidities			
Diabetes (%)	92 (26.6%)	118 (30.4%)	0.254
Hypertension (%)	185 (53.5%)	224 (57.7%)	0.252
COPD (%)	14 (4.0%)	22 (5.7%)	0.312
Atrial Fibrillation (%)	15 (4.3%)	21 (5.4%)	0.512
Malignant Neoplasms (%)	7 (2.0%)	8 (2.1%)	0.965
Previous MI (%)	24 (6.9%)	35 (9.0%)	0.315
Previous Coronary Revasc (%)	18 (5.2%)	25 (6.4%)	0.482
CKD (%)	20 (5.8%)	28 (7.2%)	0.442
Laboratory			
WBC (10^9^/L)	7.24 (5.85, 9.42)	7.55 (6.12, 9.88)	0.212
NEUT (10^9^/L)	4.85 (3.52, 6.45)	5.12 (3.88, 6.92)	0.185
Hb (g/L)	135.2 ± 14.5	132.8 ± 15.2	**0.032**
PLT (10^9^/L)	218 ± 62	215 ± 58	0.512
hsCRP (mg/L)	2.85 (1.12, 6.45)	3.52 (1.45, 7.88)	**0.012**
Cholesterol (mmol/L)	4.18 ± 0.92	4.25 ± 1.05	0.342
Triglycerides (mmol/L)	1.55 (1.12, 2.15)	1.68 (1.22, 2.35)	0.095
HDL-C (mmol/L)	1.15 ± 0.28	1.10 ± 0.25	**0.015**
LDL-C (mmol/L)	2.45 ± 0.82	2.65 ± 0.88	**0.002**
ALB (g/L)	41.85 ± 4.25	39.52 ± 4.88	**<0.001**
UA (umol/L)	342 ± 78	368 ± 85	**<0.001**
BUN (mmol/L)	4.85 (3.62, 6.45)	5.52 (4.12, 7.85)	**<0.001**
D-Dimer (mg/L)	0.28 (0.12, 0.52)	0.45 (0.22, 1.15)	**<0.001**
Angiographic			
Time to Guidewire (h)	4.52 ± 2.12	7.85 ± 3.45	**<0.001**
LAD Occlusion (%)	142 (41.0%)	205 (52.8%)	**0.001**
LCX Occlusion (%)	88 (25.4%)	102 (26.3%)	0.785
RCA Occlusion (%)	95 (27.5%)	105 (27.1%)	0.912
Bilateral/Multivessel (%)	82 (23.7%)	155 (39.9%)	**<0.001**

Bold values indicate statistical significance (*P* < 0.05).

Baseline variables were compared between patients who achieved procedural CR during the index PCI and those with incomplete revascularization in the training cohort (CR *n* = 346 vs incomplete *n* = 388). Data presentation and statistical testing are as described in Table 1.

CR, complete revascularization; LAD, left anterior descending artery; Time-to-Guidewire, symptom-to-guidewire crossing time; other abbreviations as in Table 1.

**Table 5:** Baseline characteristics stratified by procedural CR status in the testing cohort.

Variables	Complete revascularization (*n* = 165)	Incomplete revascularization (*n* = 187)	*P*-value
Demographics			
Gender (male, %)	102 (61.8%)	118 (63.1%)	0.805
Age (years)	61.85 ± 9.42	63.42 ± 10.15	0.138
Smoking (%)	40 (24.2%)	48 (25.7%)	0.758
Comorbidities			
Diabetes (%)	42 (25.5%)	55 (29.4%)	0.412
Hypertension (%)	88 (53.3%)	105 (56.1%)	0.598
COPD (%)	6 (3.6%)	10 (5.3%)	0.452
Atrial Fibrillation (%)	7 (4.2%)	11 (5.9%)	0.485
Malignant Neoplasms (%)	3 (1.8%)	4 (2.1%)	0.824
Previous MI (%)	11 (6.7%)	18 (9.6%)	0.315
Previous Coronary Revasc (%)	8 (4.8%)	12 (6.4%)	0.524
CKD (%)	9 (5.5%)	14 (7.5%)	0.448
Laboratory			
WBC (10^9^/L)	6.82 (5.42, 8.95)	7.15 (5.88, 9.42)	0.255
NEUT (10^9^/L)	4.52 (3.12, 6.15)	4.82 (3.45, 6.58)	0.182
Hb (g/L)	136.5 ± 14.2	133.2 ± 15.5	**0.038**
PLT (10^9^/L)	215 ± 58	219 ± 62	0.542
hsCRP (mg/L)	2.65 (1.05, 5.82)	3.25 (1.32, 7.42)	**0.021**
Cholesterol (mmol/L)	4.15 ± 0.88	4.22 ± 0.95	0.485
Triglycerides (mmol/L)	1.52 (1.05, 2.05)	1.65 (1.15, 2.22)	0.115
HDL-C (mmol/L)	1.16 ± 0.25	1.11 ± 0.22	**0.045**
LDL-C (mmol/L)	2.42 ± 0.75	2.62 ± 0.85	**0.022**
ALB (g/L)	42.25 ± 3.85	39.82 ± 4.52	**<0.001**
UA (umol/L)	335 ± 72	362 ± 82	**0.001**
BUN (mmol/L)	4.62 (3.45, 6.15)	5.25 (3.88, 7.42)	**0.002**
D-Dimer (mg/L)	0.25 (0.11, 0.48)	0.42 (0.21, 1.05)	**<0.001**
Angiographic			
Time to Guidewire (h)	4.32 ± 1.95	7.45 ± 3.22	**<0.001**
LAD Occlusion (%)	65 (39.4%)	98 (52.4%)	**0.015**
LCX Occlusion (%)	42 (25.5%)	50 (26.7%)	0.792
RCA Occlusion (%)	45 (27.3%)	52 (27.8%)	0.915
Bilateral/Multivessel (%)	38 (23.0%)	75 (40.1%)	**<0.001**

Bold values indicate statistical significance (*P* < 0.05).

Baseline variables were compared between patients who achieved procedural CR during the index PCI and those with incomplete revascularization in the independent testing cohort (CR *n* = 165 vs incomplete *n* = 187). Data presentation and statistical testing are as described in Table 1.

CR, complete revascularization; LAD, left anterior descending artery; Time-to-Guidewire, symptom-to-guidewire crossing time; other abbreviations as in Table 1.

In terms of laboratory profiles, the incomplete group generally showed a more adverse systemic phenotype, with higher BUN, UA, D-Dimer, and hsCRP, but lower ALB and Hb (overall trends were consistent across cohorts, with several markers reaching statistical significance), reflecting impaired renal/metabolic status, activation of the coagulation–fibrinolysis system, heightened inflammation, and poorer nutritional/synthetic status. Regarding lipid parameters, the incomplete group tended to have lower HDL-C and higher LDL-C, whereas TC and Triglycerides showed less pronounced between-group differences. For other indices, NEUT and PLT did not demonstrate a stable significant difference between groups (as shown in the tables).

#### 3.3.2 Comparison of model performance

**Figure 5:**
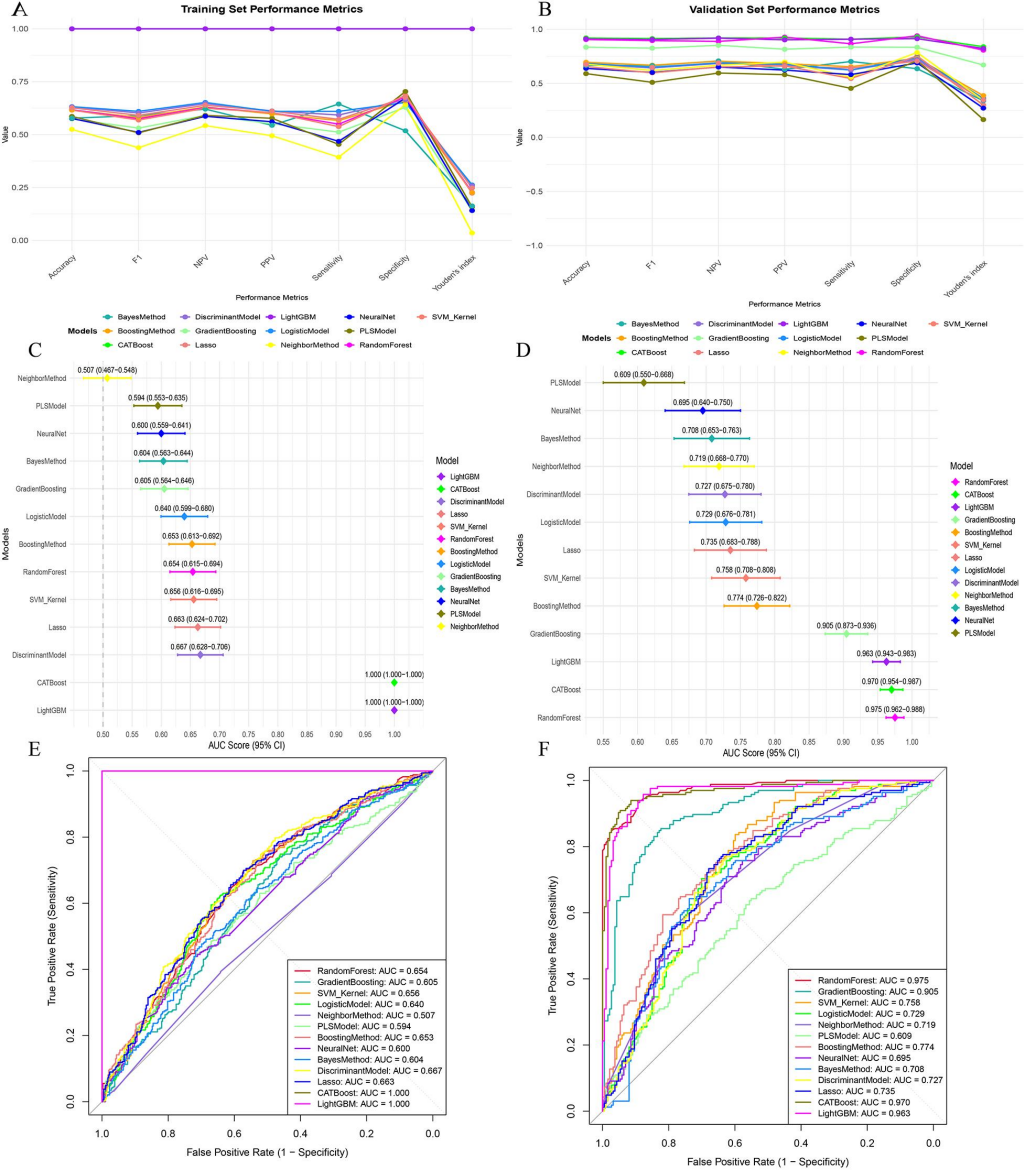
Model performance comparison for procedural CR prediction in the training and testing cohorts. **(A)** Training-cohort performance metrics across machine-learning models, including accuracy, F1-score, NPV, PPV, sensitivity, specificity, and Youden index. **(B)** Testing-cohort performance metrics across models (the “validation set” in the figure corresponds to the independent testing cohort). **(C)** Training-cohort AUCs with 95% confidence intervals across models. **(D)** Testing-cohort AUCs with 95% confidence intervals across models. **(E)** Training-cohort ROC curves across models. **(F)** Testing-cohort ROC curves across models. The corresponding metric values are labeled in the plot for clarity. CR, complete revascularization; AUC, area under the receiver operating characteristic curve; ROC, receiver operating characteristic.

For the CR prediction task, multiple ML models were compared in the training cohort and the independent testing cohort using the same framework as for the HF endpoint (Figures 5A–F). Overall, multi-metric evaluation again revealed marked heterogeneity across algorithms. In both cohorts, most models showed relatively higher specificity and NPV than sensitivity, indicating that incomplete CR and CR were not equally well identified by all candidate methods (Figures 5A,B).

In the training cohort, CatBoost and LightGBM showed the most favorable overall classification profiles across accuracy, F1-score, PPV, NPV, sensitivity, specificity, and the Youden index (Figure 5A). These two models also occupied the leading positions in the AUC forest plot and ROC analysis (Figures 5C,E). In contrast, the remaining algorithms demonstrated substantially lower discrimination, with most AUCs clustering in a clearly lower range, indicating limited ability to distinguish CR from incomplete revascularization in this dataset.

In the independent testing cohort, tree-based models continued to show clear advantages over the other candidate algorithms (Figures 5B–F). RandomForest achieved the highest discrimination (AUC = 0.975; 95% CI, 0.962–0.988), followed by CatBoost (AUC = 0.970; 95% CI: 0.954–0.987) and LightGBM (AUC = 0.963; 95% CI: 0.943–0.983). GradientBoosting also performed well (AUC = 0.905; 95% CI: 0.873–0.936), whereas the remaining models showed clearly weaker discrimination. The ROC curves similarly demonstrated that the leading tree-based models were consistently superior to the other algorithms in the testing cohort (Figure 5F).

The complete comparative performance metrics of all candidate models in the training and independent testing cohorts are additionally provided in Supplementary Table 2. Taken together, the metric comparisons, AUC forest plots, and ROC analyses supported the superiority of tree-based models for CR prediction in this cohort. Among the leading candidate models, CatBoost demonstrated the most favorable overall performance profile in the independent testing cohort and was therefore selected as the primary model for downstream evaluation. The exact comparative performance values of all candidate models in the training and independent testing cohorts are additionally provided in Supplementary Table 2.

#### 3.3.3 Clinical utility and calibration of the best-performing model (catBoost)

**Figure 6:**
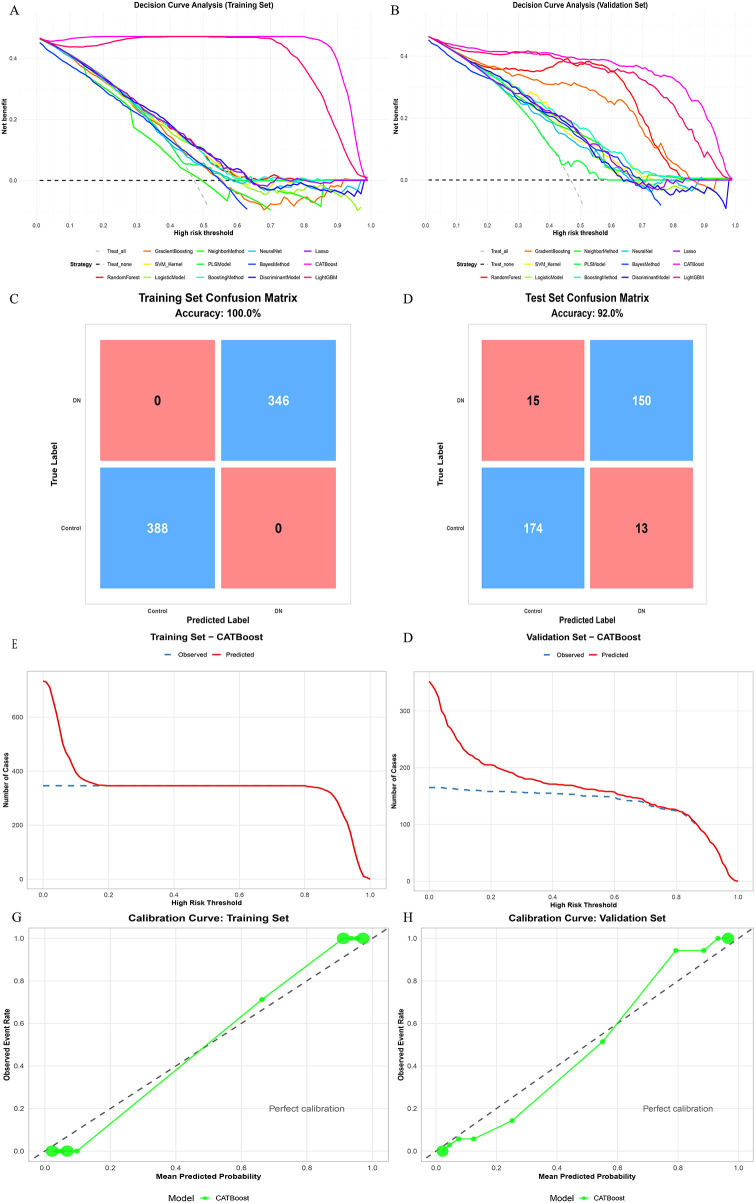
Clinical utility, classification performance, and calibration of the catBoost model for procedural CR prediction. **(A)** DCA of candidate models in the training cohort. **(B)** DCA of candidate models in the testing cohort. **(C)** Confusion matrix of the CatBoost model in the training cohort. **(D)** Confusion matrix of the CatBoost model in the testing cohort. **(E)** Clinical impact curve of the CatBoost model in the training cohort. **(F)** Clinical impact curve of the CatBoost model in the testing cohort. **(G)** Calibration curve of the CatBoost model in the training cohort. **(H)** Calibration curve of the CatBoost model in the testing cohort. CR, complete revascularization; DCA, decision curve analysis.

After selecting CatBoost as the primary model for CR prediction, we further evaluated its clinical utility and calibration in the training cohort and the independent testing cohort (Figures 6A–H). In DCA, CatBoost yielded a positive net benefit across a broad range of threshold probabilities in both cohorts and outperformed the “treat-all” and “treat-none” strategies, indicating potential value for clinical decision-making (Figures 6A,B). The confusion matrices illustrate the classification patterns of the fitted model in the training and testing cohorts (Figures 6C,D). Although the training-cohort confusion matrix showed apparent perfect classification, this result was interpreted cautiously. In the independent testing cohort, the accuracy was 92.0%, with 15 CR cases predicted as incomplete and 13 incomplete cases predicted as CR. The clinical impact curves demonstrated that, across varying high-risk thresholds, the number of individuals classified as high risk and the corresponding number of observed events followed concordant trends (Figures 6E,F). Calibration curves indicated good fit in the training cohort and overall agreement between predicted probabilities and observed event rates in the testing cohort (Figures 6G,H). Complete comparative performance metrics across candidate models in the training and independent testing cohorts are additionally provided in Supplementary Table 2.

#### 3.3.4 Explainability analysis

**Figure 7:**
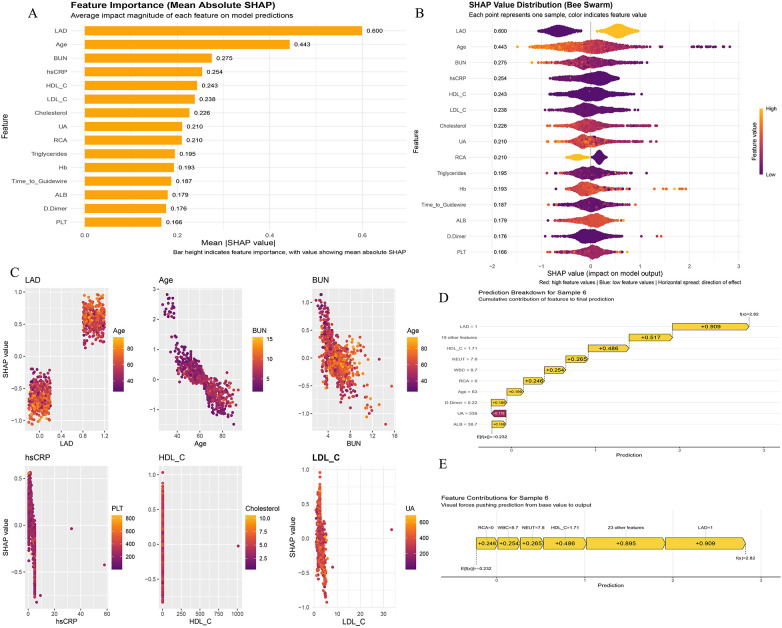
SHAP-based interpretability of the catBoost model for procedural CR prediction. **(A)** Global feature importance ranked by mean absolute SHAP values. **(B)** SHAP beeswarm plot showing the distribution and directionality of feature effects across individuals (color indicates feature value). **(C)** SHAP dependence plots for selected key predictors illustrating potentially non-linear associations with the likelihood of achieving procedural CR. **(D)** Prediction breakdown (waterfall plot) for one representative patient selected from the training cohort. **(E)** Feature contribution (force-like) plot for the same representative patient from the training cohort. SHAP, Shapley Additive Explanations; CR, complete revascularization; Time-to-Guidewire, symptom-to-guidewire crossing time.

We used SHAP to interpret the CatBoost model (Figures 7A–E). Global SHAP feature importance identified LAD, Age, BUN, hsCRP, HDL-C, LDL-C, Cholesterol, UA, RCA, Triglycerides, Hb, Time-to-Guidewire, ALB, D-Dimer, and PLT as major contributors to the model output (Figure 7A), and the SHAP beeswarm plot further illustrated the directional contributions of these variables across their value distributions to the likelihood of achieving procedural CR (Figure 7B). SHAP dependence plots suggested potentially non-linear relationships between several key predictors and model output: for example, increasing Age was generally associated with lower SHAP values, whereas LAD status showed a clear stratifying effect on the predicted likelihood of achieving CR. In addition, renal/metabolic variables (e.g., BUN and UA), inflammatory markers (e.g., hsCRP), lipid-related parameters (e.g., HDL-C, LDL-C, Cholesterol, and Triglycerides), and hematologic indices (e.g., Hb and PLT) jointly influenced the predicted probability of achieving procedural CR (Figure 7C).

For one representative patient selected from the training cohort, the waterfall plot showed that LAD involvement, HDL-C, NEUT, WBC, and RCA status were among the main contributors shifting the prediction toward a higher likelihood of CR, whereas UA and D-Dimer showed negative contributions (Figure 7D). The force-like plot provided a complementary individual-level visualization of how these features jointly moved the model output away from the baseline toward the final prediction (Figure 7E). Additional individual-level prediction breakdown and force-like plots for representative cases are provided in the Supplementary Materials.

## 4 Discussion

### 4.1 Principal findings

In this multicenter study of STEMI patients treated with PPCI, we developed and validated machine-learning prediction models for two relatively independent and clinically meaningful endpoints: (1) (in-hospital) HF during hospitalization and (2) the likelihood of achieving CR during the index PCI. By systematically benchmarking multiple algorithms in the training cohort, we identified CatBoost as the primary model for both endpoints and performed external validation in an independent testing cohort derived from the other two centers. Both models retained high discriminative performance in the testing cohort (HF: AUC ≈ 0.97; CR: AUC ≈ 0.97), supporting their generalizability. Importantly, beyond conventional discrimination metrics, we evaluated calibration and clinical utility using calibration curves, DCA, and clinical impact curves, and provided model explainability with SHAP, thereby offering a more comprehensive evidence base to support peri-procedural risk stratification and cath-lab decision support.

### 4.2 Interpretation of predictors

#### 4.2.1 Clinical interpretation and plausibility of key predictors for the HF model (CatBoost)

Based on the SHAP interpretation, the major drivers of the HF model can be broadly summarized into three domains: ischemic burden/delayed reperfusion, heightened systemic stress with inflammation–coagulation activation, and reduced physiologic reserve. First, LAD-related lesions and a longer Time-to-Guidewire (symptom-to-guidewire crossing time) were consistently associated with HF in both the training and testing cohorts, suggesting that a larger infarct territory and prolonged total ischemic time may impair left ventricular function and destabilize hemodynamics, thereby increasing the risk of (in-hospital) HF (20). Second, elevated inflammatory indices (WBC/NEUT and hsCRP) together with higher D-Dimer levels indicate intensified inflammatory response and activation of the coagulation–fibrinolysis system, which may reflect greater thrombotic burden, microvascular dysfunction, and systemic stress, ultimately contributing to deterioration in cardiac function (21, 22). Third, higher BUN and UA may reflect impaired renal function and increased metabolic burden, implying more challenging volume management and potential cardiorenal interactions; meanwhile, lower Hb, HDL-C, and ALB—representing reduced oxygen-carrying capacity, diminished metabolic/anti-inflammatory protection, and poorer nutritional/synthetic status—collectively suggest limited physiologic reserve and a reduced ability to compensate for acute myocardial injury (23, 24). Overall, these predictors align well with the pathophysiology of acute HF after STEMI and provide clinically coherent explanations for the model outputs.

#### 4.2.2 Clinical interpretation and plausibility of key predictors for the CR model (CatBoost)

Notably, Bilateral-or-Multivessel-Disease showed pronounced dominance in the initial model, suggesting that the presence of multivessel/bilateral disease is itself highly informative for whether same-session CR can be achieved (25). This is clinically plausible because multivessel disease typically implies a broader treatment scope, greater lesion complexity, longer procedural time, and higher contrast burden, which may favor a culprit-first and staged approach (26). From a modeling perspective, however, such dominance may obscure other actionable or granular signals; therefore, we excluded this feature in the primary analyses and repeated model development to evaluate robustness and interpretability without relying on a single strong indicator (initial results are shown in Supplementary Figure 3). Importantly, this model should not be interpreted as predicting operator preference in a purely subjective sense. Rather, it estimates the real-world feasibility of achieving same-session CR under the combined influence of anatomical complexity, ischemic burden, systemic condition, and procedural risk considerations.

SHAP results for the CR model suggest that the likelihood of achieving procedural CR during the index PCI is jointly influenced by (1) anatomic complexity and overall coronary disease burden, (2) total ischemic time and delays in reperfusion, and (3) systemic condition and peri-procedural risk considerations. First, LAD-related lesions and a multivessel/bilateral disease background often imply greater procedural complexity and a larger revascularization scope, potentially requiring longer procedure time and higher contrast volume, which may reduce the feasibility of completing CR within the index procedure (27, 28). Second, a longer Time-to-Guidewire (symptom-to-guidewire crossing time) generally reflects delayed care and a heavier ischemic burden, and may also coincide with less stable hemodynamics, prompting operators to prioritize culprit-vessel reperfusion and defer non-culprit interventions to a staged strategy (29). Third, inflammation-, coagulation-, renal/metabolic-, and nutrition-related markers (e.g., hsCRP, NEUT, D-Dimer, BUN, UA, ALB, and Hb) carried substantial weight in the model, indicating that heightened inflammatory stress, coagulation activation, impaired renal function, or reduced physiologic reserve may lead to a more conservative intra-procedural strategy aimed at limiting procedure duration, contrast exposure, and peri-procedural risk, thereby lowering the probability of procedural CR (30). Lipid parameters (e.g., HDL-C, LDL-C, TC, and Triglycerides) may also indirectly capture metabolic background and atherosclerotic burden, reflecting differences in lesion extent and vascular conditions that are relevant to the feasibility of achieving CR (31–33). Overall, the key predictors identified by the model are consistent with major determinants of real-world decision-making and technical feasibility for same-session complete revascularization, providing clinically coherent explanations for the model outputs.

### 4.3 Comparison with prior studies

Prior studies on peri-procedural risk in STEMI patients undergoing PPCI have largely focused on a single endpoint (e.g., in-hospital mortality, cardiogenic shock, or HF). Many of these models were derived from single-center cohorts or selected populations, and systematic reporting of external validation, calibration, and clinical utility has often been limited (34). More recently, multimodal and explainable prediction approaches have been explored in several cardiovascular settings, including post-CABG prognosis, unstable angina, and broader multicenter risk-stratification frameworks, although heterogeneity in study populations, endpoints, and validation strategies remains substantial (11, 12, 35). In contrast, our study leveraged real-world data from three centers with a training-to-external validation design, and comprehensively reported discrimination, calibration curves, DCA, and clinical impact curves, complemented by SHAP-based interpretation of key predictors—an approach aligned with emerging recommendations for standardized reporting of prediction model studies (e.g., TRIPOD + AI).

Regarding evidence for CR, randomized trials such as COMPLETE have established the benefit of a CR strategy in appropriate STEMI patients with multivessel disease by reducing ischemia-driven adverse outcomes. Subsequent trials have further explored the timing of CR (index procedure vs. staged), primarily addressing “whether” and “when” to perform CR from a strategy and prognosis perspective (4, 36). However, these studies do not directly address a pragmatic question in real-world practice: whether procedural (same-session) CR can be achieved during the index PCI, which is highly relevant to workflow management and resource planning in the catheterization laboratory. Accordingly, by modeling the likelihood of achieving CR during the index PCI as an independent prediction task—alongside (in-hospital) HF prediction—our work extends the existing literature by providing evidence oriented toward procedural feasibility assessment and cath-lab decision support.

### 4.4 Clinical implications

#### 4.4.1 Potential clinical applications of the HF prediction model

In clinical practice, (in-hospital) HF after PPCI for STEMI is closely associated with escalation of monitoring, intensification of therapy, and a higher risk of in-hospital complications. Therefore, the HF prediction model developed in this study may serve as an early peri-procedural risk stratification tool to help clinicians identify high-risk patients at admission or shortly after the procedure. For individuals predicted to be at higher risk, the model output may inform more proactive monitoring and management strategies, such as earlier bedside echocardiographic assessment, closer surveillance of volume status and hemodynamics, timely initiation or escalation of diuretics and/or inotropic support, and, when appropriate, early consideration of indications for mechanical circulatory support—potentially enabling intervention before overt clinical deterioration (37). Conversely, for patients predicted to be at low risk, the model may facilitate more efficient allocation of monitoring resources while maintaining patient safety. Importantly, this tool is intended to support risk stratification and clinical decision-making rather than replace physician judgment, and its outputs should be interpreted in conjunction with dynamic clinical course, imaging, and laboratory findings (38).

#### 4.4.2 Potential clinical applications of the CR feasibility prediction model

Unlike traditional studies that primarily address whether CR is beneficial or when staged revascularization is preferable, our CR model focuses on a pragmatic, workflow-oriented question in real-world practice: whether same-session CR is likely to be feasible during the index PCI. In the catheterization laboratory, this prediction may support procedural planning and early intra-procedural decision-making. For example, when the model indicates a high likelihood of achieving procedural CR, operators may more proactively evaluate and treat non-culprit lesions while maintaining procedural safety. Conversely, when the model suggests low feasibility of same-session CR, clinicians may consider a staged revascularization strategy in advance and tailor the procedure to minimize duration, contrast exposure, and peri-procedural risk, thereby potentially avoiding harm associated with overly prolonged interventions.

In addition, predicting CR feasibility may facilitate resource allocation and multidisciplinary coordination. For patients anticipated to require more complex interventions or longer procedural time, teams can prepare appropriate devices and support equipment, coordinate operator and monitoring/anesthesia resources, and optimize post-procedural monitoring intensity and bed planning. Importantly, the model output reflects the probability/feasibility of achieving same-session CR rather than a causal estimate of the effect of CR on outcomes. Its use should be integrated with hemodynamic status, anatomic considerations, and guideline recommendations, and it may also serve as a foundation for subsequent evaluations of prognostic differences associated with CR strategies across clinically relevant risk strata.

### 4.5 Strengths

This study has several strengths. First, we leveraged real-world data from three centers and adopted an external validation design (single-center training with independent testing from the other two centers), enabling a more reliable assessment of cross-center generalizability. Second, we addressed two complementary and clinically meaningful tasks by developing and validating separate models for (in-hospital) HF risk stratification and for estimating the feasibility of achieving CR during the index PCI, which better aligns with the continuum of peri-procedural decision-making in STEMI treated with PPCI. Third, we systematically benchmarked multiple ML algorithms within a unified framework, reducing the selection bias inherent to reporting a single model and identifying the most robust approach in the testing cohort. Fourth, beyond discrimination metrics such as AUC, we evaluated calibration and clinical utility using calibration curves, DCA, and clinical impact curves, extending model assessment from predictive accuracy to clinical usefulness. Finally, SHAP-based global and individual-level explanations clarified the key predictors and their directional contributions, enhancing transparency and clinical acceptability.

### 4.6 Limitations

Several limitations should be acknowledged. First, this was a retrospective multicenter observational study; although we performed external validation in an independent testing cohort, selection bias and unmeasured confounding cannot be fully excluded, and further confirmation in larger prospective cohorts is warranted. Second, adjudication of HF and CR primarily relied on medical records, imaging/echocardiography, and catheterization laboratory documentation; variability in definitions and documentation quality across centers may affect reproducibility and transportability. In addition, the HF endpoint was defined as an overall in-hospital HF occurrence outcome and was not further separated into HF present at admission vs. incident HF developing later during hospitalization; because these phenotypes may differ in mechanism and predictors, future studies should adopt more granular time-resolved adjudication of HF events. Third, the near-perfect performance observed for some models in the training cohort should not be overinterpreted, as it may partly reflect optimistic estimation, overfitting, or potential information leakage. Although prediction time-zero was clarified in the revised manuscript, some residual temporal ambiguity regarding retrospectively collected peri-procedural variables cannot be fully excluded. For this reason, we placed greater emphasis on discrimination, calibration, and clinical utility in the independent testing cohort, and future studies should adopt stricter time-anchored predictor definitions together with prospective multicenter validation. Fourth, we excluded patients who died in hospital, which may reduce representation of the highest-risk population and limit applicability in the most severe clinical scenarios. Finally, the models have not yet been prospectively deployed or evaluated in an impact study; thus, the real-world incremental benefit on clinical decision-making and patient outcomes remains to be determined.

### 4.7 Future directions

Future work may advance in several directions. First, both models should be independently validated in larger, geographically diverse prospective cohorts, ideally using center-stratified evaluation or leave-one-center-out validation to more rigorously assess cross-site transportability. Second, exploring parsimonious predictor subsets or developing simplified models/risk scores based on variables available earlier in the care pathway may improve bedside usability and facilitate broader implementation, with threshold strategies tailored to different resource settings. Third, integrating the models into hospital information systems or catheterization-laboratory workflows as real-time decision support tools, followed by prospective impact studies, is warranted to quantify their incremental benefits on monitoring stratification, treatment decisions, resource allocation, and clinical outcomes. Finally, extending the framework to dynamic model updating and longer-term outcome prediction (e.g., post-discharge readmission, long-term HF, or mortality) may enable continuous risk management from the acute phase through follow-up.

### 4.8 Conclusion

In summary, using multicenter clinical data from STEMI patients treated with PPCI, we developed and externally validated two prediction models: one for (in-hospital) HF risk and another for estimating the likelihood of achieving CR during the index PCI. Both models demonstrated good discrimination, acceptable calibration, and potential clinical net benefit in the independent testing cohort, supported by SHAP-based interpretability. These findings suggest that this dual-model framework may aid peri-procedural risk stratification, cath-lab strategy planning, and resource allocation, and should be further evaluated in prospective studies and real-world implementation/impact assessments.

## Data Availability

The datasets generated and/or analyzed during the current study are available from the corresponding author upon reasonable request, subject to institutional and ethical approval.

## Supplementary material

Supplementary Figure 1 Individual-level prediction breakdown plots for the CatBoost (in-hospital) HF model. Prediction breakdown (waterfall) plots are shown for 10 representative patients (Samples 1–10), illustrating how key features cumulatively move the model output from the baseline (expected) value to the final predicted risk.

Supplementary Figure 2 Individual-level feature contribution plots for the CatBoost procedural CR model. Feature contribution (force-like) plots are shown for 10 representative patients (Samples 1–10), visualizing how each feature pushes the prediction toward higher or lower probability of achieving procedural CR.

Supplementary Figure 3. Comprehensive evaluation and explainability outputs for procedural CR prediction. (A1–A2) Training/testing performance metrics across candidate models. (B1–B2) AUC forest plots with 95% confidence intervals (training/testing). (C1–C2) ROC curves (training/testing). (D1–D2) DCA curves (training/testing). (E1–E2) Confusion matrices (training/testing). (F1–F2) Clinical impact curves (training/testing). (G1–G2) Calibration curves (training/testing). (H1) Global SHAP feature importance; (H2) SHAP beeswarm plot; (H3) SHAP dependence plots for selected predictors.

Supplementary Table 1 Comparative performance of machine-learning models for in-hospital heart failure prediction in the training and independent testing cohorts.

Supplementary Table 2 Comparative performance of machine-learning models for procedural complete revascularization prediction in the training and independent testing cohorts.
