# Supplementary figures and images for: Multicenter development and validation of machine-learning risk models to predict procedural complete revascularization and in-hospital heart failure in STEMI patients treated with primary PCI
Source: Front Cardiovasc Med. 2026 May 13;13:1824937. doi: 10.3389/fcvm.2026.1824937 (PMC13212481; doi:10.3389/fcvm.2026.1824937)

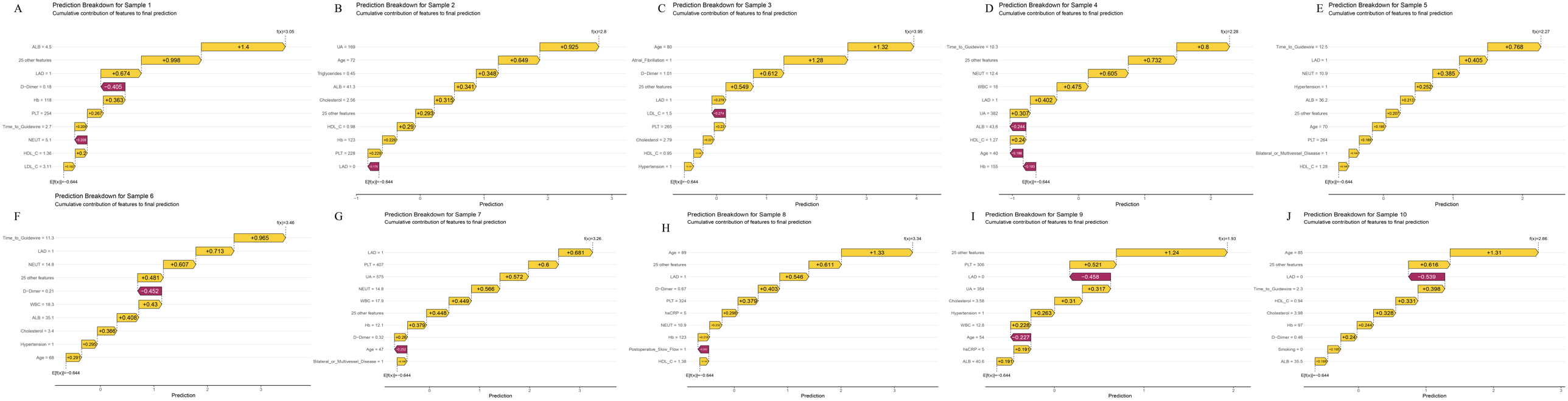

Supplement: Supplementary Figure 1 — Individual-level prediction breakdown plots for the CatBoost (in-hospital) HF model. Prediction breakdown (waterfall) plots are shown for 10 representative patients (Samples 1–10), illustrating how key features cumulatively move the model output from the baseline (expected) value to the final predicted risk. [file Image1.jpeg]

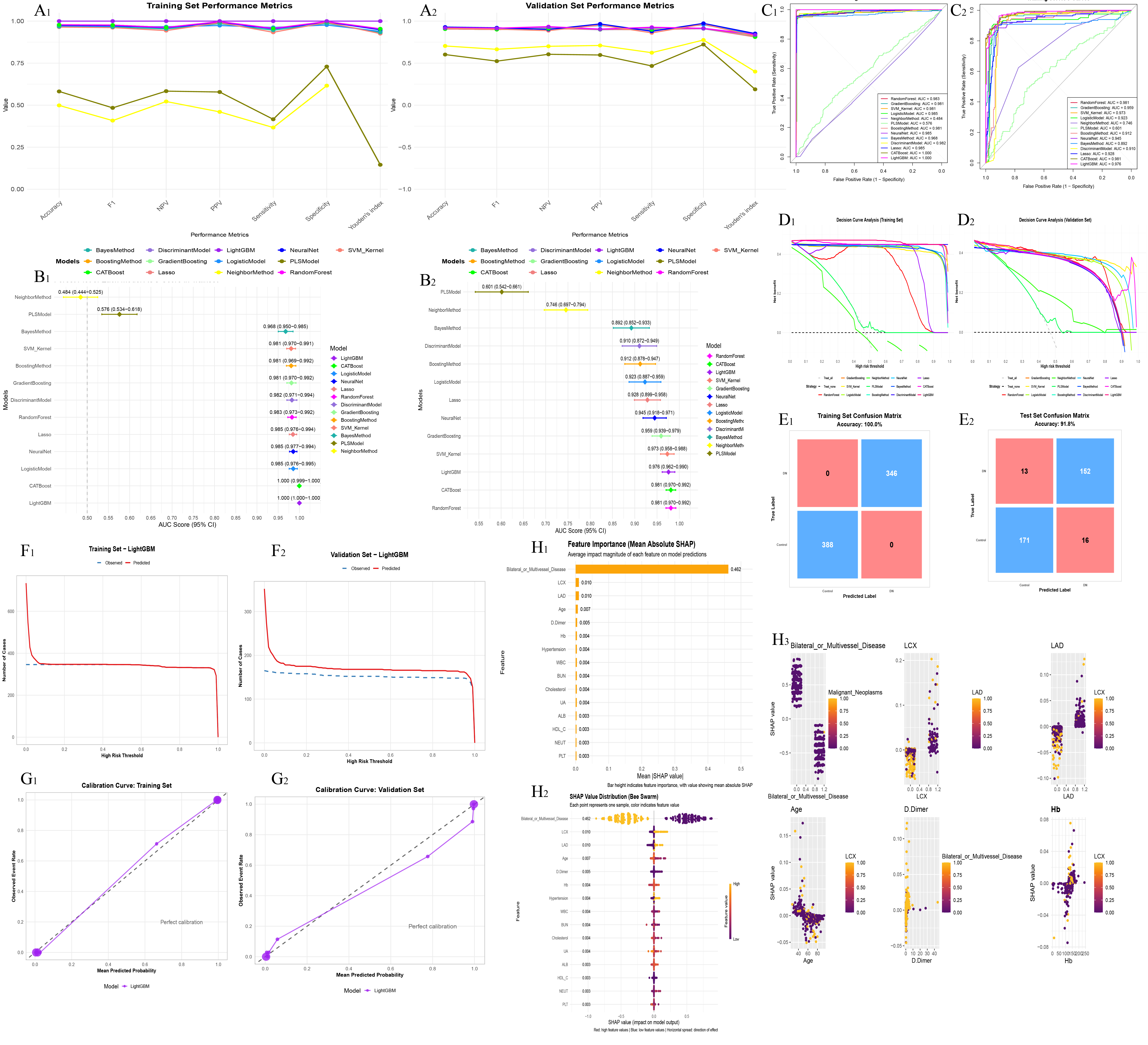

Supplement: Supplementary Figure 2 — Individual-level feature contribution plots for the CatBoost procedural CR model. Feature contribution (force-like) plots are shown for 10 representative patients (Samples 1–10), visualizing how each feature pushes the prediction toward higher or lower probability of achieving procedural CR. [file Image2.jpeg]

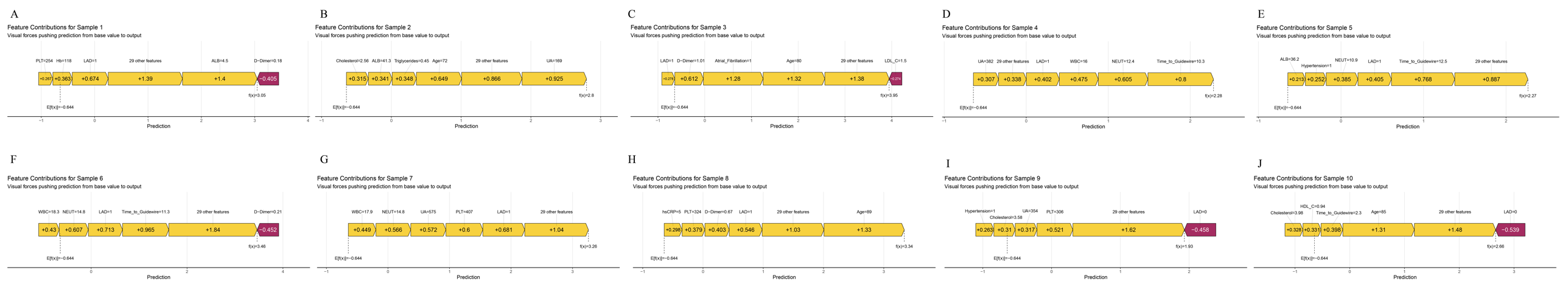

Supplement: Supplementary Figure 3. — Comprehensive evaluation and explainability outputs for procedural CR prediction. (A1–A2) Training/testing performance metrics across candidate models. (B1–B2) AUC forest plots with 95% confidence intervals (training/testing). (C1–C2) ROC curves (training/testing). (D1–D2) DCA curves (training/testing). (E1–E2) Confusion matrices (training/testing). (F1–F2) Clinical impact curves (training/testing). (G1–G2) Calibration curves (training/testing). (H1) Global SHAP feature importance; (H2) SHAP beeswarm plot; (H3) SHAP dependence plots for selected predictors. [file Image3.jpeg]
